# The PI3K/Akt Signaling Pathway Mediates the High Glucose-Induced Expression of Extracellular Matrix Molecules in Human Retinal Pigment Epithelial Cells

**DOI:** 10.1155/2015/920280

**Published:** 2015-01-28

**Authors:** Dong Qin, Guo-ming Zhang, Xun Xu, Li-ya Wang

**Affiliations:** ^1^Henan Eye Institute, Henan Provincial Eye Hospital, Zhengzhou, Henan 450003, China; ^2^Shenzhen Key Laboratory of Ophthalmology, Shenzhen Eye Hospital and Jinan University, Shenzhen 518040, China; ^3^Department of Ophthalmology, Shanghai First People's Hospital, Shanghai Jiaotong University, Shanghai 200080, China

## Abstract

Prolonged hyperglycemia is an important risk factor of the pathogenesis of diabetic retinopathy (DR). Extracellular matrix molecules, such as fibronectin, collagen IV, and laminin, are associated with fibrotic membranes. In this study, we investigated the expression of fibronectin, collagen IV, and laminin in RPE cells under high glucose conditions. Furthermore, we also detected the phosphorylation of protein kinase B (Akt) under high glucose conditions in RPE cells. Our results showed that high glucose upregulated fibronectin, collagen IV, and laminin expression, and activated Akt in RPE cells. We also found that pretreatment with LY294002 (an inhibitor of phosphatidylinositol 3-kinase) abolished high glucose-induced expression of fibronectin, collagen IV, and laminin in RPE cells. Thus, high glucose induced the expression of fibronectin, collagen IV, and laminin through PI3K/Akt signaling pathway in RPE cells, and the PI3K/Akt signaling pathway may contribute to the formation of fibrotic membrane during the development of DR.

## 1. Introduction

Diabetic retinopathy (DR) is a major cause of adult blindness globally [1]. Prolonged hyperglycemia plays a vital role in the development of DR. Increased synthesis of extracellular matrix molecules contributes to the thickening of the basement membrane, which is common in DR [2]. Proliferative DR, an advanced stage of DR, is characterized by epiretinal outgrowth of fibrotic membranes at the vitreoretinal interface. Retinal pigment epithelial (RPE) cells, which are located between the neurosensory retina and the vascular choroids, form the outer blood-retinal barrier and play an important role in the pathological processes that lead to the loss of vision. The breakdown of the outer blood-retinal barrier can activate RPE cells, which then initiate proliferation and migration and secrete extracellular matrix molecules to combat certain diseases, such as proliferative vitreoretinopathy (PVR) [3], proliferative DR [4], and age-related macular degeneration (AMD) [5]. The main components of the fibrotic membranes are extracellular matrix molecules, which are combined with some cell types. It has been confirmed that RPE and other cell types contribute to the formation of fibrotic membranes [6, 7].

The PI3K/AKT pathway is important not only in the development of many diseases but also for signaling in normal cells. This pathway plays a key role in numerous cellular functions, including adhesion, proliferation, migration, invasion, metabolism, and survival [8]. The PI3K pathway also plays an essential role in the formation of normal blood vessels [9]. The PI3K/Akt signaling pathway is required for the insulin-dependent regulation of cellular and systemic metabolisms [10]. In addition to insulin, cytokines, growth factors, and environmental stresses can activate the PI3K/Akt signaling pathway, mainly for regulating cell proliferation, motility, differentiation, and survival [11, 12]. The activation of the PI3K/Akt signaling pathway, which is mediated through molecular aberrations, is instrumental in promoting the development of tumors as well as in resisting anticancer therapies [13, 14].

It has been reported that high glucose can activate the PI3K/Akt signaling pathway in podocytes [15], vascular smooth muscle cells [16], vascular endothelial cells [17], and human pancreatic cancer cells [18] and that it can induce the expression of extracellular matrix molecules in human renal proximal tubular cells [19]. In this study, we investigate the high glucose-induced expression of extracellular matrix molecules (such as fibronectin, collagen IV, and laminin), the phosphorylation of Akt, and the mechanism involved in the high glucose-induced expression of fibronectin, collagen IV, and laminin in RPE cells.

## 2. Methods

### 2.1. Reagents

Anti-fibronectin and anti-collagen IV were obtained from Abcam (Danvers, MA). Anti-laminin was purchased from Novus. Anti-human GAPDH was obtained from Bioworld Technology, Inc. LY294002 was purchased from Sigma (St. Louis, MO). p-Akt and total-Akt were obtained from Cell Signaling Technology (Danvers, MA).

### 2.2. Cell Culture

Human RPE cells (ARPE-19; CRL-2302) were purchased from the American Type Culture Collection (ATCC; Manassas, VA, USA). The cells were cultured in Dulbecco's Modified Eagle Medium (DMEM; Gibco, Invitrogen, Grand Island, NY) with 10% fetal bovine serum (FBS, Gibco, Invitrogen), 100 units/mL penicillin, and 100 *μ*g/mL streptomycin (Sigma, St. Louis, MO) at 37°C under 5% CO_2_ and 95% ambient air. RPE cells were seeded in a 25 cm^2^ flask at a density of 3 × 10^6^.

### 2.3. Gene Expression Analysis by Real-Time PCR

Human RPE cells were plated in 6-well culture dishes and incubated until 80% confluence was reached. During pretreatment, the cells were incubated with LY294002 for 12 h and then in 5.6 mM (normal glucose, NG) or 25 mM (high glucose, HG) glucose for an additional 24 h. The total RNA was extracted from RPE cells using TRIzol reagent (Invitrogen, Carlsbad, CA) according to the manufacturer's instructions. cDNA was synthesized with 2 *μ*g of total RNA using a RevertAid First Strand cDNA Synthesis Kit (Fermentas). The real-time PCR assays were performed using IQ Supermix (Bio-Rad, Hercules, CA), and each 20 *μ*L reaction mixture contained 2 *μ*L cDNA, 10 *μ*L SYBR Green Real-Time PCR Master Mix, 7.2 *μ*L sterilized water, and 0.8 *μ*L of each primer (10 *μ*M). Amplification was performed in 96-well plates using an iCycler iQ real-time detection system (Bio-Rad). The PCR primers were as follows: human fibronectin: forward 5′-GAT AAA TCA ACA GTG GGA GC-3′, reverse 5′-CCC AGA TCA TGG AGT CTT TA-3′; human collagen IV: forward 5′-AGA GTC AGC ATC GGC TAC CT-3′, reverse 5′-AGG AAG GGC ATG GTG CTG AA-3′; human laminin: forward 5′-CTA AGC TGG CTC CCG ATG-3′, reverse 5′-CAG GAA GAG CAG CAG AAC CT-3′; human GAPDH: forward 5′-TGT TCG ACA GTC AGC CGC AT-3′; reverse 5′-ACT CCG ACC TTC ACC TTC CC-3′. The thermocycling conditions were 3 min at 94°C to activate the iTaq DNA polymerase, 39 cycles of 20 s each, 94°C for denaturation, 20 s at 61°C for annealing, and 20 s at 72°C for extension. The mRNA expression was normalized to the expression level of GAPDH and was calculated using the following equation: Fold change = 2^−ΔΔCT^.

### 2.4. Western Blot Analysis

Human RPE cells were grown to confluence in 6-well culture dishes and were incubated for pretreatment with LY294002 for 12 h and then in NG or HG glucose for an additional 24 h. Western blot was performed as described previously [20, 21]. In brief, cells were harvested and lysed in RIPA lysis buffer containing PMSF protease inhibitors. The protein concentrations for each sample were determined using the bicinchoninic acid assay (BCA). Protein samples were analyzed on 6% or 10% SDS-PAGE gels, transferred to PVDF membranes (Millipore, Billerica, MA), and processed for analysis using an enhanced chemiluminescence (ECL) detection system (Amersham, Arlington Heights, IL). The primary antibodies were used at the following dilutions: anti-fibronectin (1 : 1,000), anti-collagen IV (1 : 1,000), anti-laminin (1 : 1,000), anti-p-Akt (1 : 2000), anti-total Akt (1 : 1000), and anti-GAPDH (1 : 4,000).

### 2.5. Immunofluorescence Staining

To detect fibronectin, collagen IV, and laminin, the cells on glass coverslips were fixed with 4% paraformaldehyde in phosphate-buffered saline (PBS; 20 min), washed (3x, PBS), and 0.4% triton X100 (15 min). Blocking was performed with 10% goat serum (1 h, 23°C). Primary antibodies were diluted into 10% goat serum/PBS and incubated overnight at 4°C. The following antibodies were used: rabbit anti-fibronectin (1 : 100), rabbit anti-collagen IV (1 : 50), and rabbit anti-laminin (1 : 200). Secondary antibodies were used, TRITC conjugated goat anti-rabbit IgG (1 : 200). The samples were counterstained with DAPI (1 : 1000; No. D9542, Sigma, USA).

### 2.6. Statistical Analysis

The statistical analysis was performed using SPSS software (version 17.0; SPSS, Inc., Chicago, IL). It used a one-way analysis of variance and a Student's *t*-test. A *P* value of <0.05 was considered to be statistically significant. Data are expressed as means ± standard deviation (SD).

## 3. Results

### 3.1. Induction of Fibronectin, Collagen IV, and Laminin mRNA and Protein Expression under High Glucose Conditions

**Figure 1:**
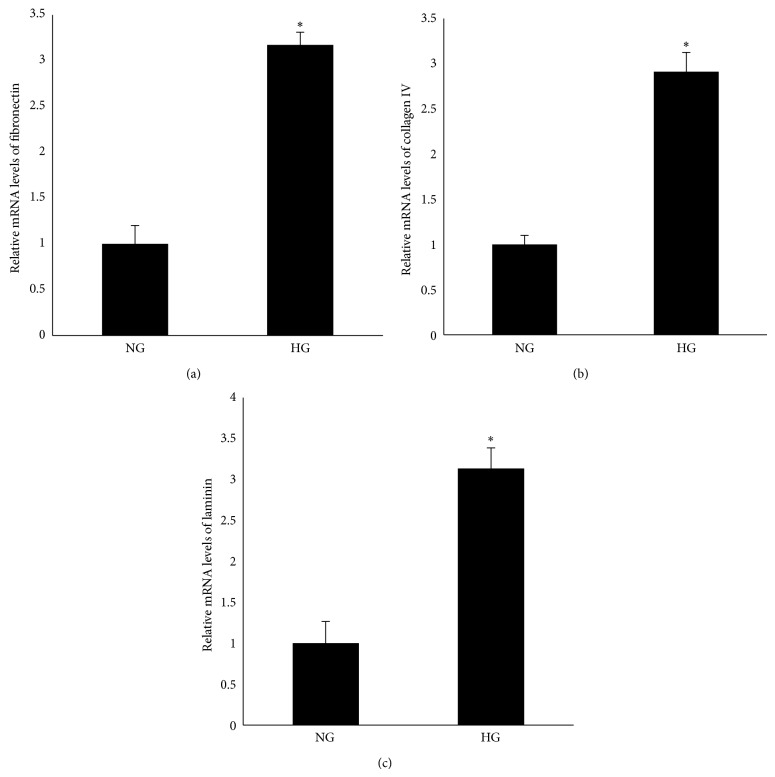
High glucose induced the mRNA expression of fibronectin, collagen IV, and laminin in RPE cells. RPE cells were exposed to NG (5.6 mM) or HG (25 mM) for 24 h before measuring the mRNA expression of fibronectin, collagen IV, and laminin. (a, b, c) Compared with NG, a real-time PCR analysis showed that the mRNA expression of fibronectin, collagen IV, and laminin was upregulated in response to HG. The data shown represent the mean ± SD of three independent experiments. ^*^ *P* < 0.01 versus NG.

**Figure 2:**
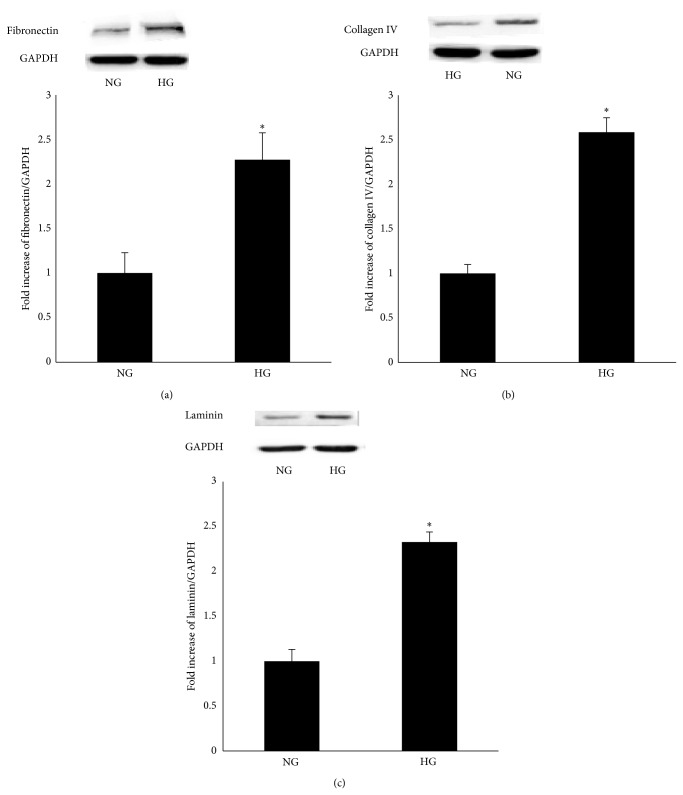
Effect of a high glucose concentration on the expression of fibronectin, collagen IV, and laminin protein in RPE cells. (a, b, c) A western blot analysis demonstrated that the protein expression of fibronectin, collagen IV, and laminin was upregulated when exposed to high glucose conditions for 24 h in RPE cells. NG and HG represent the normal glucose group and the high glucose group. The data represent the mean ± SD of three independent experiments. ^*^ *P* < 0.01 versus NG.

To examine the effect of high glucose on the expression of fibronectin, collagen IV, and laminin, RPE cells were cultured in Dulbecco's Modified Eagle Medium containing either 5.5 mM (NG) or 25 mM (HG) and were exposed for 24 h. Real-time PCR data revealed increased mRNA levels of fibronectin, collagen IV, and laminin in the cells in the 25 mM medium (Figures 1(a)–1(c)). A western blot analysis showed that an increased protein level was also observed in the cells in the 25 mM medium (Figures 2(a)–2(c)).

### 3.2. Immunofluorescence Staining of Fibronectin, Collagen IV, and Laminin in RPE Cells

**Figure 3:**
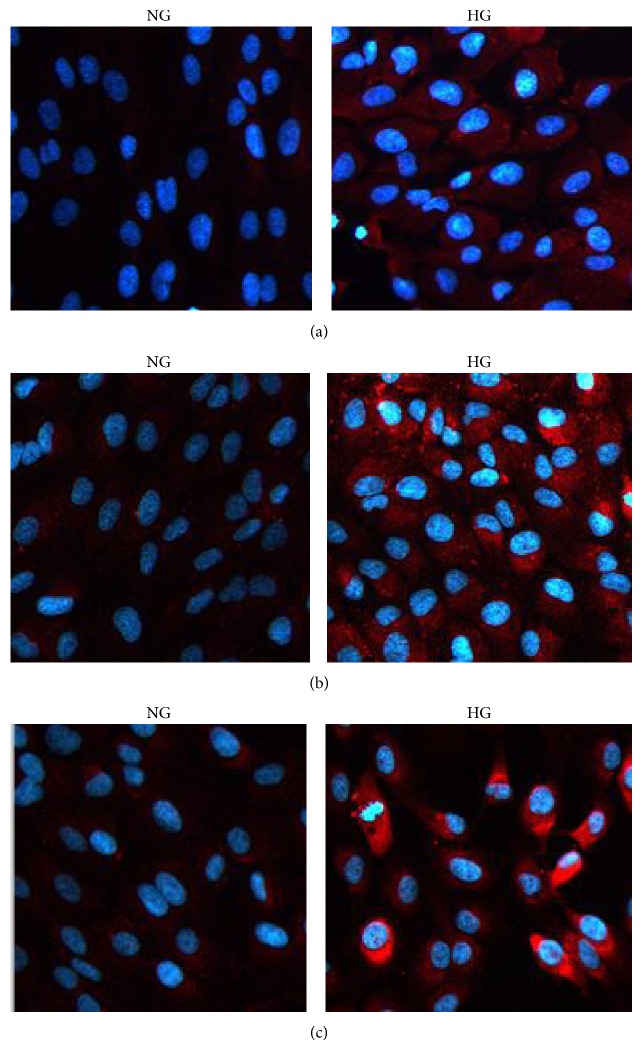
Immunofluorescence evaluation of fibronectin, collagen IV, and laminin in RPE cells. The expression of fibronectin, collagen IV, and laminin was evaluated after incubation with 24 h in RPE cells under high glucose conditions. The RPE cells showed more intense staining under high glucose conditions compared with NG. (a) fibronectin; (b) collagen IV; (c) laminin. DAPI: blue.

Under normal condition, fibronectin, collagen IV, and laminin staining were weak in RPE cells. However, under high glucose condition, strong positive staining of fibronectin, collagen IV, and laminin were observed in the cytosol and nucleus in RPE cells (Figures 3(a)–3(c)). After pretreatment with LY294002, positive staining of fibronectin, collagen IV, and laminin were inhibited under high glucose conditions (Figures 7(a)–7(c)).

### 3.3. High Glucose Activates PI3K/Akt Signaling Pathway in RPE Cells

**Figure 4:**
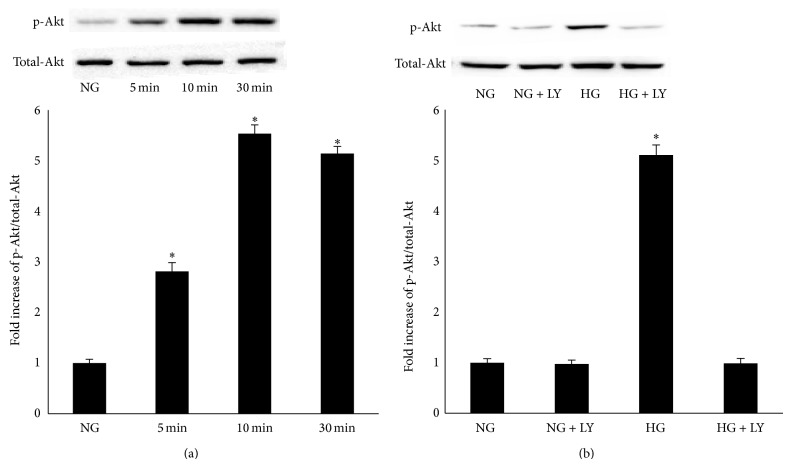
(a) Activation of Akt by high glucose in RPE cells. PRE cells were stimulated with high glucose (25 mM) for the indicated periods of time, and cell lysates were immunoblotted with anti-p-Akt and anti-Akt antibodies. ^*^ *P* < 0.001 versus NG. (b) RPE cells were pretreatment with 20 *μ*M LY294002 for 30 min and then incubated with high glucose for 30 min for assay of Akt phosphorylation. Levels of phosphorylated Akt were determined with western blot analysis. The data represent the mean ± standard deviation (SD) of three independent experiments. ^*^ *P* < 0.001 versus NG or HG + LY.

To investigate whether high glucose activates Akt phosphorylation, RPE cells were cultured in Dulbecco's Modified Eagle Medium containing either 5.5 mM (NG) or 25 mM (HG) and were exposed for 5 min, 10 min, or 30 min. A western blot analysis showed that high glucose can activate the phosphorylation of Akt in RPE cells (Figure 4(a)). The phosphorylation of Akt was blocked by pretreatment with LY294002 under high glucose condition (Figure 4(b)).

### 3.4. PI3K/Akt Signaling Pathway Mediates the Expression of Fibronectin, Collagen IV, and Laminin in RPE Cells under High Glucose Conditions

**Figure 5:**
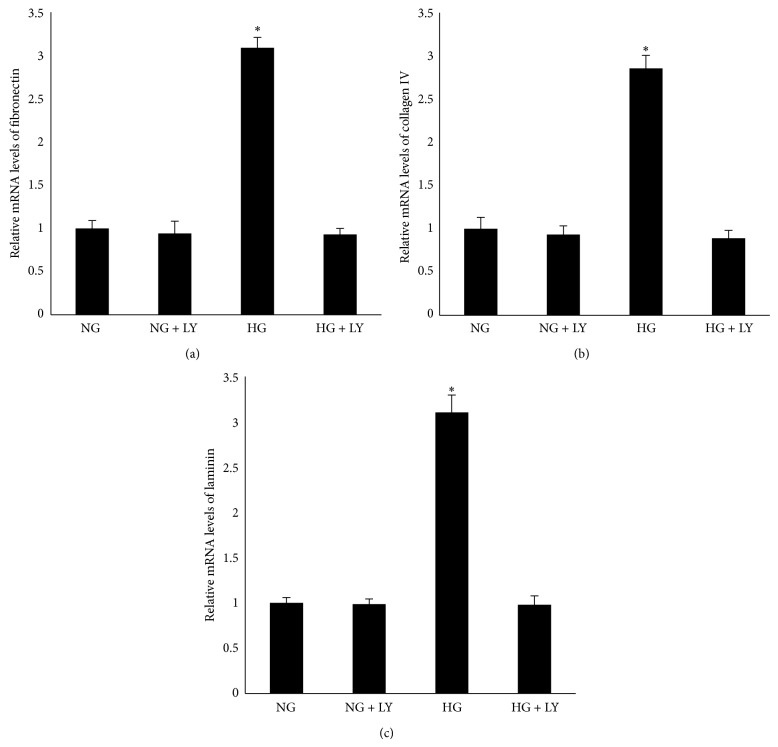
The PI3K/Akt signaling pathway mediated the mRNA expression of fibronectin, collagen IV, and laminin in RPE cells under high glucose conditions. (a, b, c) After pretreatment with 20 *μ*M LY294002 for 12 h, the mRNA expression of fibronectin, collagen IV, and laminin was significantly decreased when exposed to high glucose conditions for 24 h in RPE cells. ^*^ *P* < 0.01 versus NG or HG + LY. The data shown represent the mean ± SD of three independent experiments.

**Figure 6:**
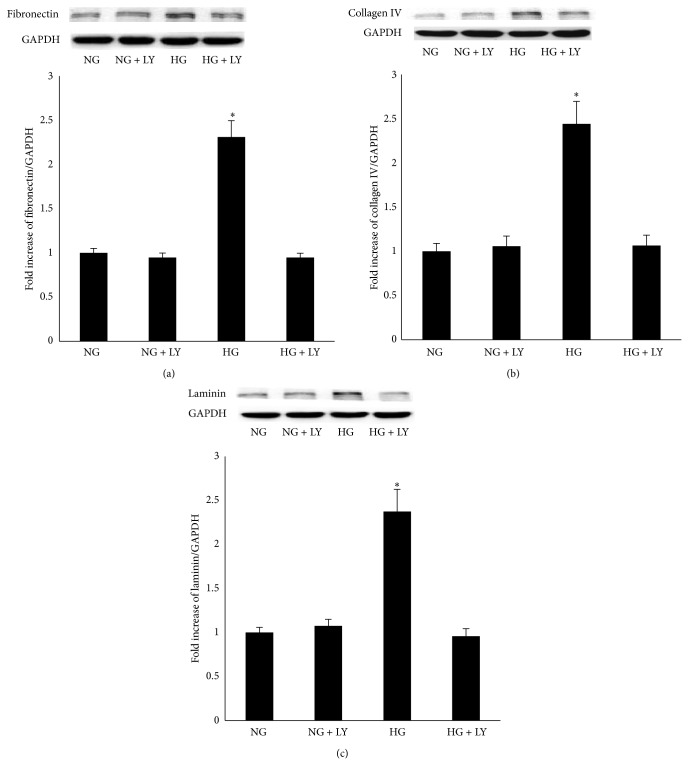
The protein level of fibronectin, collagen IV, and laminin was mediated by PI3K/Akt signaling pathway in RPE cells under high glucose conditions. (a, b, c) The protein expression of fibronectin, collagen IV, and laminin was significantly decreased when exposed to high glucose conditions for 24 h in RPE cells by pretreatment with 20 *μ*M LY294002 for 12 h. ^*^ *P* < 0.01 versus NG or HG + LY. The data shown represent the mean ± SD of three independent experiments.

**Figure 7:**
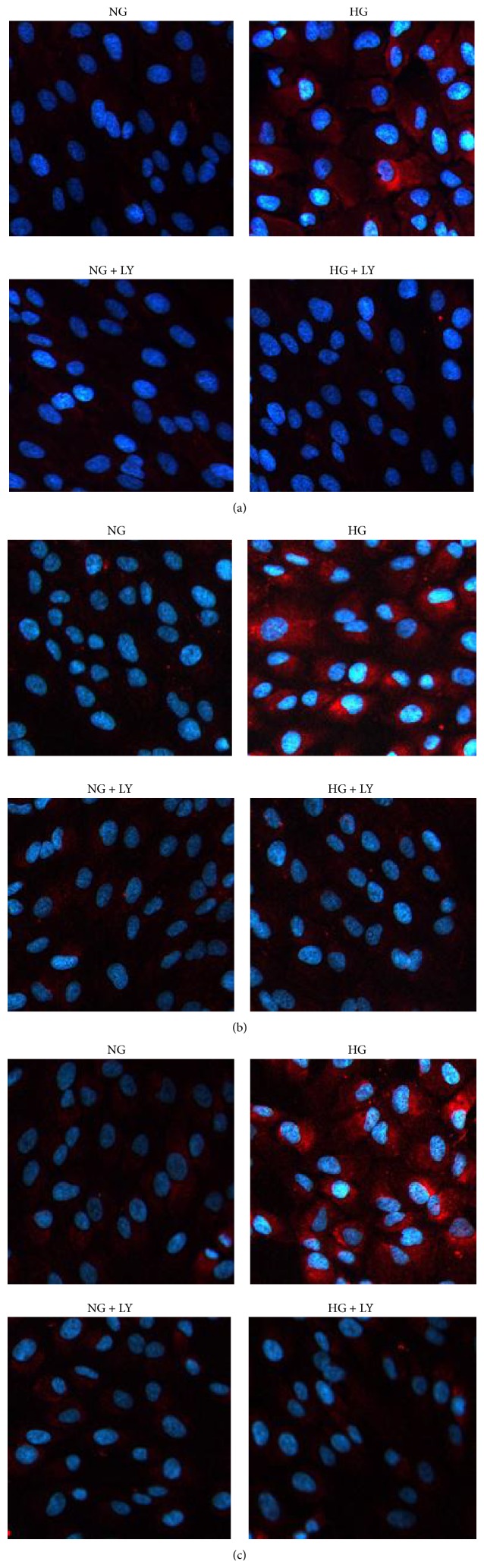
Immunofluorescence staining of fibronectin, collagen IV, and laminin in RPE cells. The expression of fibronectin, collagen IV, and laminin was evaluated by pretreatment with 20 *μ*M LY294002 for 12 h and then incubation with 24 h in RPE cells under high glucose conditions. The RPE cells showed more intense staining under high glucose conditions compared with NG or HG + LY. (a) Fibronectin; (b) collagen IV; (c) laminin. DAPI: blue.

Having found that high glucose activated the PI3K/Akt signaling pathways and induced the expression of fibronectin, collagen IV, and laminin in RPE cells, we examined whether the activation of the PI3K/Akt signaling pathways plays a vital role in the high glucose-induced expression of fibronectin, collagen IV, and laminin in RPE cells. Using a real-time PCR assay, pretreatment of RPE cells with LY294002 blocked the high glucose-induced expression of fibronectin, collagen IV, and laminin mRNA (Figures 5(a)–5(c)). A western blot analysis also showed that the high glucose-induced protein levels of fibronectin, collagen IV, and laminin were blocked by pretreatment with LY294002 in RPE cells (Figures 6(a)–6(c)).

## 4. Discussion

Hyperglycemia is one of the most important risk factors for the development of DR. Sustained hyperglycemia can activate many factors, cytokines, and other molecules. The expression of extracellular matrix molecules was detected in many organs of diabetes patients and cell types under high glucose conditions [22–25]. It has also been proven that extracellular matrix molecules are involved in the formation of fibrotic membranes and the thickening of basement membrane during the development of DR [26]. In the present study, we demonstrated that, compared with the control group, high glucose significantly increased the induction of extracellular matrix molecules (fibronectin, collagen IV, and laminin) in RPE cells when exposed to 25 mM glucose for 24 h. However, information about the link between high glucose and extracellular matrix molecules remains unclear.

To gain further insight into the molecular mechanisms by which high glucose induces the expression of fibronectin, collagen IV, and laminin in RPE cells, we examined intracellular signaling pathways. Our results showed that the phosphorylation of Akt was activated when exposed to high glucose at 5 min, 10 min, and 30 min in RPE cells. It has been reported that sustained endothelial activation of Akt induces the formation of structurally and functionally abnormal blood vessels that recapitulate the aberrations of tumor vessels [27]. Studies have also demonstrated that the inhibition of Akt signaling could inhibit pathological vascularization [28]. Inhibition of PI3K/Akt signaling should be considered especially in cancer therapy because the inappropriate activation of this pathway is frequently observed in many tumor types [29]. This study showed that the expression of fibronectin, collagen IV, and laminin was significantly decreased when pretreated with LY294002 for 12 h and then exposed to high glucose conditions for an additional 24 h in RPE cells.

Fibrosis is defined as the excessive deposition of extracellular matrix molecules into organs and tissues. Extracellular matrix molecules and RPE cells contribute to the formation of fibrotic membranes in proliferative DR [6, 7, 26]. The present study demonstrated that high glucose significantly promoted the induction of extracellular matrix molecules and the phosphorylation of Akt under high glucose conditions in RPE cells. In addition, inhibition of the PI3K/Akt signaling pathway significantly decreased the expression of extracellular matrix molecules under high glucose conditions in these cells. Thus, the PI3K/Akt signaling pathway may contribute to the formation of fibrotic membranes during the development of DR.
